# Anxiolytic-Like Effects of Bergamot Essential Oil Are Insensitive to Flumazenil in Rats

**DOI:** 10.1155/2019/2156873

**Published:** 2019-08-14

**Authors:** Laura Rombolà, Damiana Scuteri, Annagrazia Adornetto, Marilisa Straface, Tsukasa Sakurada, Shinobu Sakurada, Hirokazu Mizoguchi, Maria Tiziana Corasaniti, Giacinto Bagetta, Paolo Tonin, Luigi Antonio Morrone

**Affiliations:** ^1^Department of Pharmacy, Health and Nutritional Sciences, Section of Preclinical and Translational Pharmacology, University of Calabria, 87036 Rende, Italy; ^2^First Department of Pharmacology, Daiichi College of Pharmaceutical Sciences, 815-8511 Fukuoka, Japan; ^3^Department of Physiology and Anatomy, Tohoku Pharmaceutical University, 981-8558 Sendai, Japan; ^4^Department of Health Sciences, University “Magna Graecia” of Catanzaro, 88100 Catanzaro, Italy; ^5^Regional Center for Serious Brain Injuries, S. Anna Institute, Crotone, Italy

## Abstract

Anxiety disorders are one of the most common mental disorders, and benzodiazepines (BDZs), acting on gamma-aminobutyric acid type A (GABA-A) receptor complex, represent the most common antianxiety medications in the world. However, chronic BDZ use elicits several adverse reactions. Reportedly, aromatherapy is safer for the management of anxiety. Bergamot essential oil (BEO) extracted from *Citrus bergamia* Risso *et* Poiteau fruit, like other essential oils, is widely used in aromatherapy to relieve symptoms of stress-induced anxiety. Interestingly, preclinical data indicate that BEO induces anxiolytic-like/relaxant effects in animal behavioural tasks not superimposable to those of benzodiazepine diazepam. To better elucidate the involvement of GABAergic transmission, the present study examines the effects of pretreatment with flumazenil (FLZ), a benzodiazepine site antagonist, on BEO effects using open-field task (OFT) in rats. The data yielded show that FLZ does not significantly affect behavioural effects of the phytocomplex. These results demonstrate the lack of overlapping between BEO and BDZ behavioural effects, contributing to the characterization of the neurobiological profile of the essential oil for its rational use in aromatherapy.

## 1. Introduction

Several essential oils extracted from fruits belonging to *Citrus* genus are used in aromatherapy to treat anxiety, sleep, mood, and cognitive disorders [1, 2]. It is hypothesized that bergamot originates from *Citrus aurantium* L. and *Citrus limon* L. or *Citrus aurantifolia* Swing [3]. Bergamot essential oil (BEO) is obtained by cold pressing of the epicarp and, partly, of the mesocarp of the fresh fruit of *Citrus bergamia* Risso *et* Poiteau, according to the Italian Pharmacopeia XII ed. BEO comprises a volatile fraction (93–96% of total) containing oxygenated derivatives (such as linalool) and sesquiterpene and monoterpene hydrocarbons (such as limonene) and a nonvolatile fraction (4–7% of total) containing polymethoxylated flavones, waxes, coumarins, and psoralens such as bergamottin (5-geranyloxypsoralen) and bergapten (5-methoxypsoralen) [4, 5]. Limonene, *γ*-terpinene and *β*-pinene, together with linalool, and linalyl acetate are among the most abundant compounds found in the volatile fraction and all together constitute >90% of the whole oil [6, 7]. In the last decade, preclinical studies have supported the therapeutic use of BEO. Particularly, bergamot oil is endowed with remarkable neurobiological effects [8, 9] partially deriving from an interference with basic mechanisms finely tuning synaptic plasticity under physiological [10, 11] and pathological conditions, i.e., brain ischemia [12], pain [13–18], and behavioural and psychological symptoms of dementia [19, 20]. Under basal condition, BEO elevates extracellular levels of discrete amino acids with neurotransmitter function after systemic or focal administration in the hippocampus of freely moving rats [10]. Moreover, systemic doses of BEO increase the electrocorticographic correlate of alert, wakefulness, and relaxation [11]. The capacity of BEO to modify EEG frequency, related with alert and relaxation, supports data showing anxiolytic-relaxant effects after systemic [21] or inhaled [22] administration of this phytocomplex in animal behavioural tasks. Interestingly, controversial results are obtained comparing the effects of BEO with those of diazepam (DZP). In fact, while Saiyudthong and Marsden speculate that active components of the phytocomplex interact with BDZ site on GABA-A receptor complex inducing anxiolytic effects [22], we have recently demonstrated the properties of BEO do not overlap with the effects of DZP [21]. Since GABAergic transmission plays a key role in the pathophysiology of anxiety disorders, to gain more insight regarding the mechanism involved in anxiolytic-like effects of BEO, the aim of the present study is to investigate the effects of flumazenil, an antagonist at BDZ binding site, on the behaviour induced by BEO in the open-field test (OFT) in rats.

## 2. Materials and Methods

### 2.1. Animals

Male Wistar rats (250–300 g; Charles River, Calco, Italy) were housed in groups of four in standard laboratory cages (40 × 25 × 15 cm) at constant temperature (22 ± 1°C) and relative humidity (50%) under a regular light-dark schedule (lights on 7 a.m. to 7 p.m.). Before the OFT experiment, rats were allowed one week of adaptation to the laboratory conditions with free access to food and water. The European Community Council Directive of 24 November 1986 (86/609/EEC) and L.D. 4 March 2014 No. 26 has been followed to minimize the number of animals used still generating reliable results.

### 2.2. Materials

BEO was kindly provided by “Capua Company1880 S.r.l.,” Campo Calabro, Reggio Calabria (Italy). Chromatographic results on the certificate of analysis provide the following composition of the batch: *d*-limonene, 39.60%; linalyl acetate, 31.09%; and linalool, 9.55%. FLZ was purchased from Tocris Cookson, Inc. (Bristol, UK), dissolved in Tween80 and diluted with 0.9% NaCl (final concentration of Tween80 was 2%) [23, 24], and was freshly prepared on the day of the experiment.

### 2.3. Experimental Procedure

The acclimatization of the rats was performed for 2 h on the day of the experiment. The animals were subjected to habituation. Random experimental groups were formed. The rats underwent intraperitoneal (i.p.) administration of BEO (500 *µ*L/kg) [10, 11, 21] or jojoba oil [14–16, 21]. I.p. of FLZ (3 mg/kg), reverting BDZ effects in OFT [25, 26], or its vehicle (tween in saline solution) was administered as pretreatment 15 minutes earlier. After thirty minutes, behavioural effects were evaluated by using the OF test [27]. This experimental protocol is conformed to literature data where pretreatment with FLZ reverts behavioural effects of different natural products in OFT in rodents [25, 26].

The rat was observed from a screen in a near laboratory since the test was recorded with a closed-circuit camera. Behavioural tests were carried out between 09.00 and 13.00 a.m. according to the circadian rhythms. Video recordings were subjected to the following examination by a blinded (unaware of the treatments) observer. An overdose of isoflurane was used to euthanize the animal after the experiment. Behavioural room was ventilated, and the arena was washed with water and cleaned up with 70% ethanol after daily sessions to avoid any remaining odour traces of BEO and excrements.

### 2.4. Open-Field Test

The open-field test is considered as an indicator of the emotional state of the animal, which allows an easy and fairly rapid assessment of well-defined behaviours, such as crossing, wall rearing, grooming, center, and immobility, and it is widely used in the screening of drugs that act on the central nervous system (CNS) [27]. Particularly, crossing and wall rearing can be, respectively, regarded as indicators of locomotor activity and exploratory behaviour, whereas grooming, center, and immobility are behaviours positively correlated with fear or emotionality [27–29]. The rat was placed in the center of a dark plastic circular arena (75 cm diameter) in a sound proof room with 20 lux light [27]. The duration of each behavioural session was 20 min. The assessed parameters (general locomotor activity in an interval of 5 min (square limit crossings with both forepaws and wall rearing), anxiety-related behaviour (face and body grooming, head washing, and time spent in the center of arena), and immobility) were videotaped and analyzed to be scored (Labehaviour).

### 2.5. Statistics

Statistical analysis consisted of one-way or two-way ANOVA, followed by Tukey's multiple comparisons test using Graph Pad® 6.0. *p* value <0.05 was considered statistically significant.

## 3. Results

**Figure 1:**
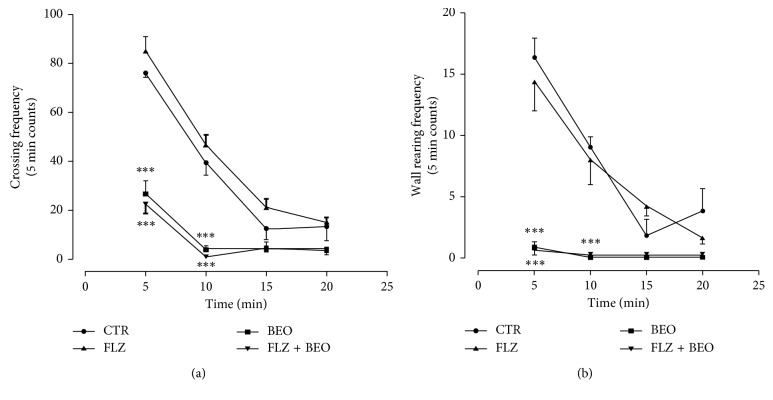
Crossing and wall rearing frequency in open-field test in male Wistar rats after systemic (i.p.) administration (500 *µ*l/kg) of Tween80 in saline + jojoba oil (CTR), FLZ (3 mg/kg) + jojoba oil (FLZ), Tween80 in saline + BEO (BEO), and FLZ (3 mg/kg) + BEO (FLZ + BEO). Data are expressed as mean ± SEM of total frequency counts in 5 min (*n* = 5 per group). ^*∗∗∗*^*p* < 0.0001 vs. CTR group (two-way ANOVA, followed by Tukey's multiple comparisons test).

Two-way ANOVA analysis indicates differences between treatment and time regarding the frequencies of crossing and wall rearing (Table 1). In particular, BEO administration reduces both behaviours reaching statistical significance when compared to the CTR group (Figure 1). Systemic administration of FLZ, 15 min before BEO, does not modify the decrease in frequencies of crossing and wall rearing measured in rats treated with the essential oil (Figure 1).

**Figure 2:**
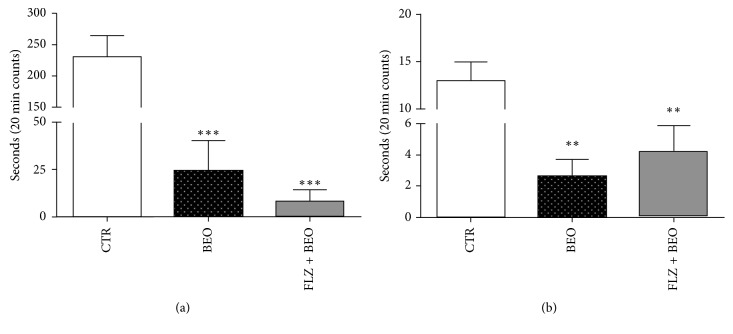
Grooming and time spent in center of arena in the open-field test in male Wistar rats after systemic (i.p.) administration of saline + jojoba oil (CTR), Tween80 in saline + BEO (BEO), and FLZ (3 mg/kg) + BEO (FLZ + BEO). Data are expressed as mean ± SEM of seconds (*n* = 5 per group). ^*∗∗*^*p* < 0.001, ^*∗∗∗*^*p* < 0.0001 vs. CTR group (one-way ANOVA, followed by Tukey's multiple comparisons test). (a) Grooming. (b) Center.

One-way ANOVA analysis also indicates differences in grooming, center, and immobility time (Table 2). A statistically significant decrease is observed for grooming in the animals treated with BEO versus CTR group. Pretreatment with FLZ is not able to prevent this effect (Figure 2).

In regard to the period in the middle of the arena, the results indicate that systemic administration of BEO reduces this behaviour reaching statistical significance when compared to the CTR group (Figure 2). Pretreatment with FLZ does not influence this effect of BEO although it is shown a slight trend (*p* > 0.05) toward an increase of the time spent in the center (Figure 2).

**Figure 3:**
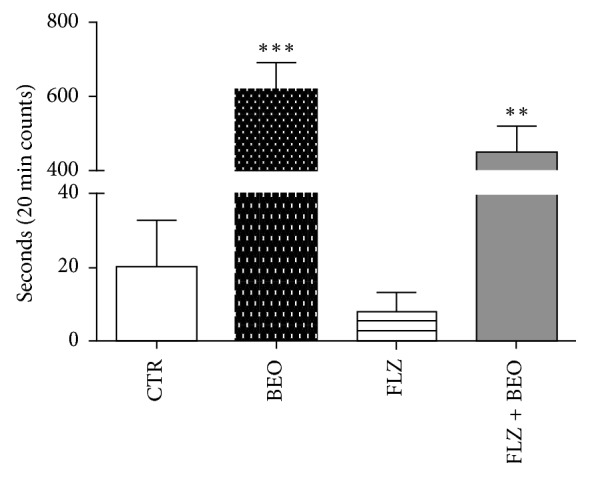
Immobility in the open-field test in male Wistar rats after systemic (i.p.) administration of Tween80 in saline + jojoba oil (CTR), FLZ (3 mg/kg) + jojoba oil (FLZ), Tween80 in saline + BEO (BEO), and FLZ (3 mg/kg) + BEO (FLZ + BEO). Data are expressed as mean ± SEM of seconds (*n* = 5 per group). ^*∗∗*^*p* < 0.001, ^*∗∗∗*^*p* < 0.0001 vs. CTR group (one-way ANOVA, followed by Tukey's multiple comparisons test).

Furthermore, the animals treated with BEO, although still vigilant, spend more time in immobility compared to the control group (Figure 3). Interestingly, in the animals pretreated with FLZ, it is observed a trend to decrease immobility observed in the BEO group, though no statistical difference is calculated (*p* > 0.05) (Figure 3).

For all the parameters studied, pretreatment with FLZ in the control group does not affect animal behaviours in the OFT supporting literature data using a wide dose range of 0.1–10 mg/kg [30–32].

## 4. Discussion

BEO, like other essential oils, is widely used in aromatherapy to minimize symptoms of stress-induced anxiety though the underlying mechanism and its interference on GABAergic transmission remain to be elucidated [21, 22].

Administration of BEO exerts anxiolytic-like effects in OFT not significantly counteracted by FLZ, a BDZ site antagonist. The latter findings confirm that the properties of BEO do not parallel those of BDZs [21]. Here we have observed that locomotor activity and exploratory behaviour, measured by crossing and wall rearing frequencies, are reduced in the animals treated with BEO compared to the vehicle group and pretreatment with FLZ failed to prevent the effects of the phytocomplex. Moreover, in the 20-minute OFT session, it is measured a reduction in grooming behaviour by BEO that is not affected by pretreatment with FLZ. Altogether, these results indicate that BDZ receptor is not involved in these effects of BEO. Pretreatment with FLZ partially prevents the effects of BEO on immobility and time spent in center of the arena, i.e., behaviours positively correlated with fear or emotionality, though this did not reach statistical significance. Interestingly, the latter would support an involvement of GABA-A receptor in the effects of BEO on these behaviours. GABA-A receptor contains the isosteric site for the endogenous ligand and several different allosteric binding sites, which modulate the activity of the receptor indirectly and are the targets of various synthetic and natural compounds [33, 34]. Molecules with different chemical structures and affinities affect the binding site and exhibit distinct pharmacological effects. Particularly, several pharmacological actions can be attributed to the modulation of specific alpha subunits [35]. For example, sedative and hypnotic properties are mediated by GABA-A receptors containing alpha-1 subunits, whereas positive modulation of GABA-A receptors at alpha-2 and/or alpha-3 subunits displays anxiolytic effects [36].

Interestingly, we previously demonstrated that systemic administration of BEO induces a moderate release of GABA in the hippocampus of freely moving rats [10]. This neurochemical finding, together with the effects of FLZ on immobility and center reported here, could support the hypothesis that some of the compounds present in the phytocomplex might interfere with GABA-A receptor mediated transmission. Several studies have investigated the involvement of this neurotransmission in the anxiolytic-like effects of single volatile compounds present in essential oils such as BEO. Particularly, the authors suggest that administration route of the compounds could modulate their action on BDZ site affecting anxiolytic effects. For example, linalool, administered by inhalation, induces anxiolytic effects prevented by FLZ [24], while systemic administration does not produce anxiolytic effects [37]. Furthermore, systemic administration of a mixture of *cis* and *trans* (+)-limonene epoxide induces anxiolytic effects that are prevented by pretreatment with FLZ [38]. However, when the (R)-(+)-limonene is administered by inhalation, anxiolytic effect is not blocked by the BDZ site antagonist [39].

Other neurotransmitter systems have been implicated in the anxiolytic-like effects induced by essential oils. For instance, mice subjected to inhalation of lemon oil present an antistress effect through modulation of dopaminergic, serotonergic, and GABAergic neurotransmission [40]. Inhalation of lavender essential oil shows anxiolytic effects increasing the level of serotonin (5-HT) in the prefrontal cortex, striatum [41], and hippocampus [42]. Moreover, WAY100635, a 5-HT receptor antagonist, blocks the action of the essential oil of lavender while the use of antagonists of GABA-A receptor is devoid of effect [42, 43]. Similar data are also achieved by Galdino et al. and by Tabari et al. who have observed anxiolytic effects after systemic injection of *Spiranthera odoratissima* and *Rose geranium* essential oils counteracted by pretreatment with 5-HT1A receptor antagonists but not by FLZ [44, 45].

Altogether, these data support the hypothesis that the anxiolytic-like effects of essential oils and likely of BEO may be underlined by multiple complex mechanisms that deserve further investigation.

## 5. Conclusion

BDZs are well-developed and commonly prescribed drugs used to treat anxiety disorders; however, several side effects (e.g., lethargy, dizziness, drowsiness, sedation, and dependence) can be severe, so development of new drugs is necessary. These adverse reactions have prompted the spread of aromatherapy, though limited data are available about the mechanisms underlying anxiolytic activity of the essential oils. The results of our study contribute to deepen the characterization of the neuropharmacological profile of BEO. The latter lend support to the hypothesis that the anxiolytic-like effects of this phytocomplex are not superimposable to those of BDZs and to its aromatherapeutic use for the treatment of stress-induced anxiety.

## Figures and Tables

**Table 1:** Crossing and rearing.

Crossing			Rearing		
*F* (DFn, DFd) treatment	*F* (DFn, DFd) time	*F* (DFn, DFd) time × treatment	*F* (DFn, DFd) treatment	*F* (DFn, DFd) time	*F* (DFn, DFd) time × treatment
*F* (3, 64) = 91.99	*F* (3, 64) = 122.5	*F* (9, 64) = 12.76	*F* (3, 64) = 54.76	*F* (3, 64) = 31.01	*F* (9, 64) = 9.29
*p* < 0.0001	*p* < 0.0001	*p* < 0.0001	*p*=0.0001	*p* < 0.0001	*p*=0.0001s

Degree of freedom from between the columns (DFn), degree of freedom from within the columns (DFd), and *p* values in two-way ANOVA considering treatment and time.

**Table 2:** Grooming, center, and immobility.

Grooming	Center	Immobility
*F* (DFn, DFd)	*F* (DFn, DFd)	*F* (DFn, DFd)
*F* (2, 12) = 32.09	*F* (2, 12) = 11.91	*F* (3, 16) = 35.59
*p* < 0.0001	*p*=0.0014	*p* < 0.0001

Degree of freedom from between the columns (DFn), degree of freedom from within the columns (DFd), and *p* values in one-way ANOVA considering treatment.

## Data Availability

The data used to support the findings of this study are available from the corresponding author upon request.
